# Thoracic endovascular aortic repair with true-false-true lumen deployment

**DOI:** 10.1016/j.jvscit.2020.03.005

**Published:** 2020-05-28

**Authors:** Neel A. Mansukhani, Matthew C. Chia, Gabriel A. Wallace, Andrew W. Hoel, Mark K. Eskandari

**Affiliations:** a Division of Vascular Surgery, Department of Surgery, Northwestern University, Chicago, Ill; b Division of Vascular and Endovascular Surgery, Department of Surgery, Medical College of Wisconsin, Milwaukee, Wisc

**Keywords:** Aortic dissection, Type B dissection, Thoracic aneurysm, TEVAR, False lumen

## Abstract

Endovascular treatment of aortic dissection may be complicated by challenges to navigating the true lumen. In this report, we describe treatment of a type B dissection after open type A repair with aneurysmal degeneration, a short-segment occluded true lumen, and a distal re-entry tear near the celiac artery origin. Endovascular septal fenestration and subsequent thoracic endovascular aortic repair were used to bypass the short-segment midthoracic aortic occlusion, successfully excluding the thoracic aortic aneurysm. The patient was discharged without complications, and follow-up imaging demonstrated favorable aortic remodeling. The case demonstrates feasibility of an endovascular bypass of an intervening short-segment occluded true lumen using a thoracic endovascular aortic repair with true-false-true lumen deployment.

Thoracic endovascular aortic repair (TEVAR) has become the first-line treatment of descending thoracic aortic diseases, such as aortic dissection, aortic aneurysm, and traumatic aortic injury.^1^, ^2^, ^3^ Compared with open surgical treatment, TEVAR offers favorable perioperative morbidity and mortality,^4^^,^^5^ although device-specific instructions for use specify indication (eg, dissection, aneurysm) and proximal and distal landing zone diameters in native, normal aortic true lumen. Given the minimally invasive nature of TEVAR, off-label endovascular techniques have frequently been reported for unique clinical settings, particularly for complex aortic dissections; these include false lumen intentional placement (FLIP),^6^^,^^7^ the knickerbocker technique,^8^ the candy-plug technique,^9^ and other adjuncts.^10^ In this report, we describe the treatment of a chronic type B aortic dissection complicated by aneurysmal degeneration and short-segment occlusion of an intervening midthoracic aortic true lumen with endovascular bypass using septal fenestration followed by TEVAR. The patient consented to the publication of his case and images.

## Case report

**Fig 1:**
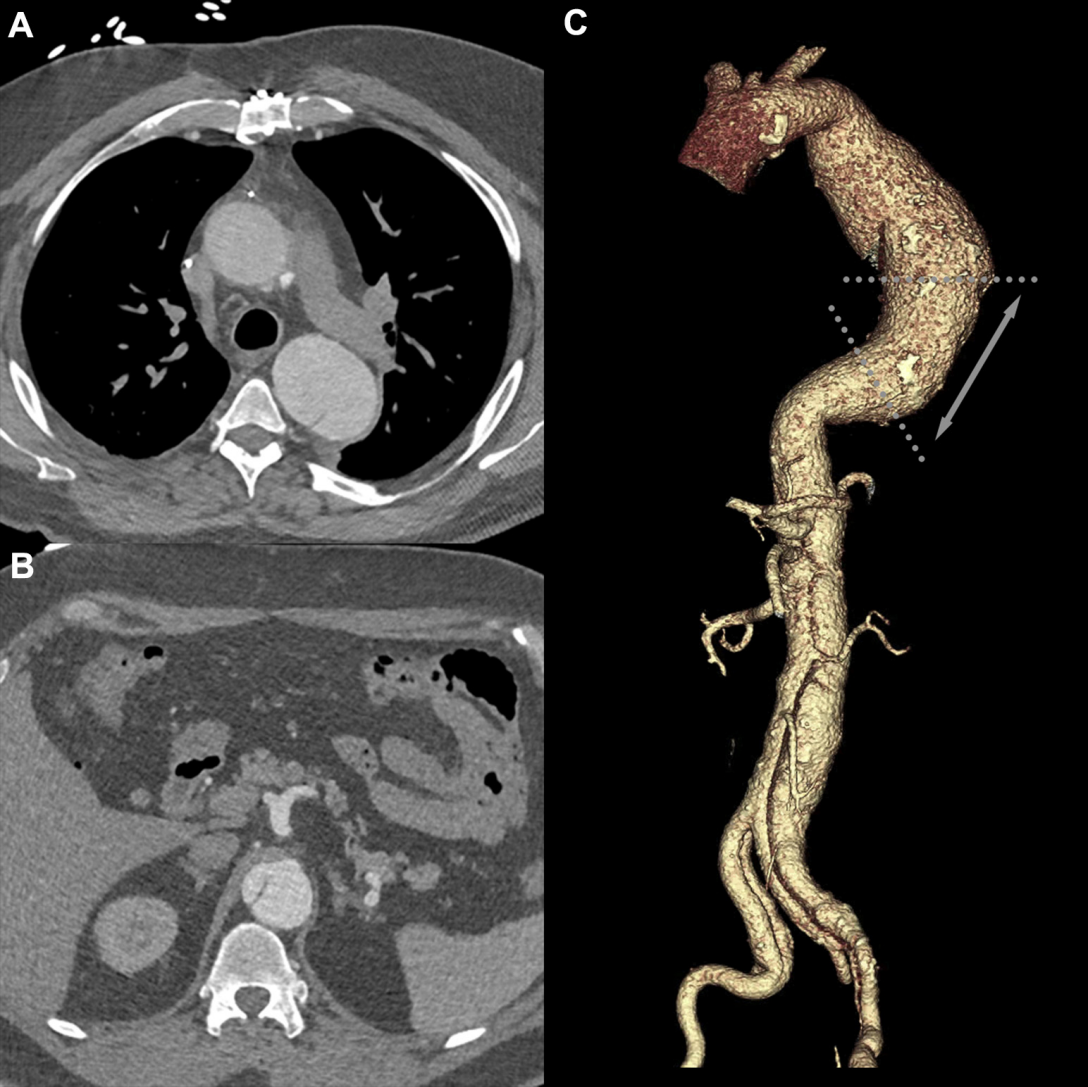
Computed tomography angiogram of a 47-year-old man with aneurysmal degeneration of a chronic type B aortic dissection with a short-segment midthoracic aortic occluded true lumen. **A,** Proximal extent of dissection with initial entry tear. **B,** Distal re-entry tear 5 mm cephalad to the celiac artery origin. **C,** Three-dimensional reconstruction of dissection; *dotted lines* represent extent of true lumen occlusion.

**Fig 2:**
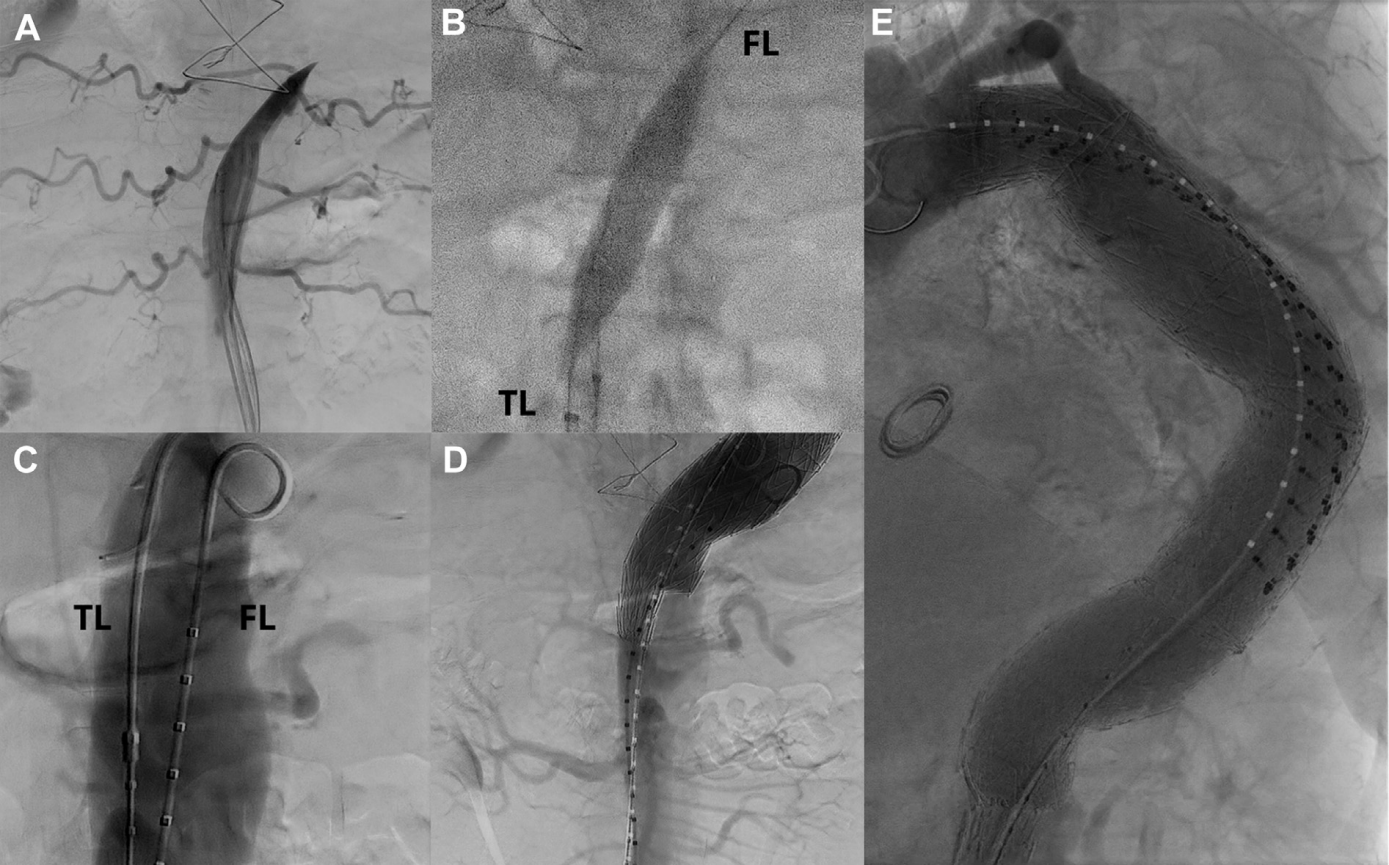
Fluoroscopic views of aortic dissection flap endovascular fenestration. **A,** Angiogram of the short-segment midthoracic occluded true lumen with filling of intercostal arteries by retrograde flow. **B,** Balloon dilatation of endovascularly created fenestration from true lumen (*TL*) into false lumen (*FL*). **C,** Post-fenestration angiogram with filling of both TL and FL. **D,** Completion angiogram of distal seal zone with filling of mesenteric and renal arteries without evidence of retrograde flow into the aneurysm. **E,** Completion angiogram showing arch vessels and exclusion of thoracic aneurysm without evidence of retrograde flow.

A 47-year-old man with a history of acute Stanford type A dissection requiring open ascending aortic repair and subsequent need for redo aortic valve replacement presented to vascular surgery clinic with aneurysmal degeneration of a residual chronic type B aortic dissection. His prior repair consisted of a Bentall with hemiarch without need for total arch replacement, and there was no evidence of residual arch dissection at presentation. The anatomy of the type B dissection was as follows: a large proximal entry tear distal to the left subclavian artery (Fig 1, *A*), a distal re-entry tear proximal to the celiac axis (Fig 1, *B*), and a maximum aneurysm diameter of 6.2 cm. The dissection extended through the visceral segment into the right common and left external iliac arteries, with the superior mesenteric, renal, and inferior mesenteric arteries arising off of the true lumen and the celiac artery arising off of the false lumen. The aneurysm was limited to the descending thoracic aorta, with adequate access vessels and a proximal seal zone in native, nondissected aorta of at least 20 mm in length. The proximity of the distal re-entry tear to the origin of the celiac artery (<5 mm) raised concern about the risk of a stent-induced new entry tear as well as possible dissection into the celiac artery. Therefore, the decision was made to land the endograft in the true lumen cephalad to the distal re-entry tear. In the midthoracic aorta, the true lumen appeared to be either severely compressed or occluded on preoperative computed tomography angiography (Fig 1, *C*). During the initial TEVAR attempt, a short-segment intervening occlusion of the thoracic aorta was confirmed on diagnostic aortography. Unsuccessful attempts were made to traverse the short-segment occlusion with hydrophilic guidewires and catheters. This occlusion was located in zone 3 over a short distance, and the true lumen in zone 4 was preserved by retrograde flow from the distal re-entry tear (Fig 2, *A*). Because of the patient's comorbid disease burden (chronic kidney disease, heart failure, obstructive sleep apnea, atrial fibrillation, obesity), two prior sternotomies, and prior aortic surgery, an endovascular approach was favored.

The procedure was performed under general anesthesia with cerebrospinal fluid drainage, arterial pressure monitoring, bilateral percutaneous ultrasound-guided common femoral arterial access with ProGlide preclose percutaneous closure (Abbott, Santa Clara, Calif), and systemic heparinization. Through the left-sided access, a hydrophilic Glidewire and Glidecath (Terumo, Somerset, NJ) were used to select the true lumen to the level of the occluded true lumen proximal to the celiac artery (Fig 2, *A*). Through the right-sided access, the false lumen was selected through the natural fenestration proximal to the celiac artery and confirmed with intravascular ultrasound (IVUS). The natural fenestration was balloon dilated with 16-mm XXL (Boston Scientific, Marlborough, Mass) and 25-mm Tyshak (B. Braun Medical, Bethlehem, Pa) balloons. However, it was determined that there was inadequate length to land an aortic endograft in the true lumen between the fenestration and the origin of celiac artery. Therefore, from the left-sided access, a Rösch-Uchida transjugular intrahepatic portosystemic shunt kit (Cook Medical, Bloomington, Ind) was advanced through the true lumen up to the level of the most proximal portion of the reconstituted true lumen in the midthoracic aorta. Under IVUS guidance (right-sided access), the aortic dissection septum was crossed using the Rösch-Uchida (left-sided access). Wire access was thus established from the common femoral true lumen to the level of the newly created aortic fenestration, across the dissection septum into the false lumen in the mid-descending thoracic aorta, through the proximal entry tear back into the true lumen at zone 3, and into the aortic arch. This was confirmed with angiography and IVUS. The newly created septal fenestration was balloon dilated with a 16-mm XXL balloon (Fig 2, *B*), with post-septoplasty angiography demonstrating opacification of both lumens (Fig 2, *C*).

**Fig 3:**
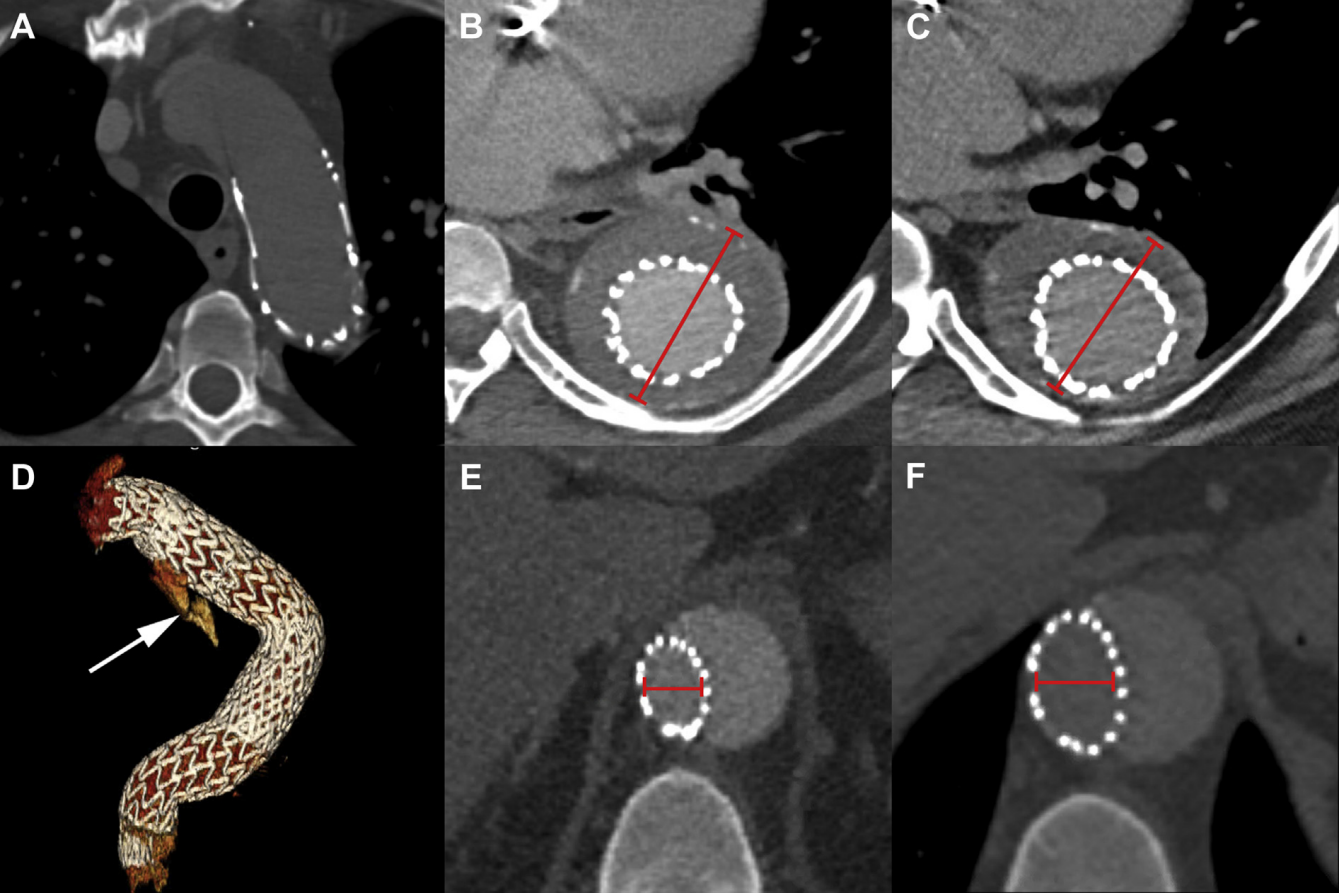
Follow-up computed tomography angiograms. **A,** Proximal seal zone at 6 months. **B,** Maximum diameter at 1 week measures 6.2 cm. **C,** Maximum diameter at 6 months measures 5.6 cm. **D,** Three-dimensional reconstruction of repair; *arrow* notes type II endoleak. **E,** Distal seal zone at 1 week measures 15 mm. **F,** Distal seal zone at 6 months measures 17 mm.

Next, three sequential Gore C-TAG devices (W. L. Gore & Associates, Newark, Del) were deployed, effectively excluding the aneurysm and bypassing the occluded portion of the aortic true lumen and re-establishing in-line true lumen aortic circulation (37-mm × 10-cm proximal descending thoracic aorta, 37-mm × 20-cm mid-descending thoracic aorta, 37-mm × 20-cm distal descending thoracic aorta). The distal portion of the most distal device that crossed the newly created aortic fenestration was gently dilated using a 25-mm Tyshak balloon. Completion angiography demonstrated patency of the stent graft, preservation of arch and visceral vessels, and exclusion of the aneurysm without evidence of endoleak including any retrograde flow that might suggest a type IB leak (Fig 2, *D* and *E*). IVUS was then performed to evaluate the ostia of the mesenteric vessels, which were free from obstruction. Wires and catheters were removed, access vessels were closed without complication, and heparin was reversed with systemic protamine administration. The patient was extubated, with normal neurologic examination findings on conclusion of the procedure, and he was discharged home on postoperative day 4. On follow-up computed tomography angiography performed at 1 week, 1 month, and 6 months postoperatively, the stent grafts were in good position without migration (Fig 3, *A*) with decreased aneurysm sac size from 6.2 cm to 5.6 cm (Fig 3, *B* and *C*) and no evidence of a type IB endoleak. There was an intercostal artery-based type II endoleak (Fig 3, *D*), but the endograft at the distal seal zone had expanded from 15 mm to 17 mm, suggesting remodeling of the chronic aortic dissection septum (Fig 3, *E* and *F*).

## Discussion

TEVAR has become the most common method for treatment of diseases of the thoracic aorta. In comparison to both optimal medical therapy and open surgical repair, TEVAR decreases perioperative morbidity and mortality, although midterm and late outcomes remain unclear.^11^, ^12^, ^13^ Traditional open surgical techniques are still advocated in some circumstances, especially in patients with associated connective tissue disease and those who are young and fit for surgery.^1^ Despite this, instructions for use for all TEVAR devices do not advise placement outside of the true lumen in a type B aortic dissection. In this case, we demonstrate successful exclusion of aneurysmal degeneration of a chronic type B aortic dissection with an intervening occluded true lumen. Our intentional TEVAR placement in the false lumen was able to successfully exclude the thoracic aneurysm from retrograde flow from the false lumen. However, authors have described a need for adjunctive techniques, such as candy-plug embolization^9^ and the knickerbocker technique,^8^ which fully exclude a false lumen in the thoracic region. We were prepared to pursue these options after deployment of the endograft, but completion angiography revealed successful exclusion of the aneurysm.

Data on selection of patients for FLIP of TEVAR devices and the outcomes of patients who undergo this repair are limited. Quinones-Baldrich et al^6^^,^^14^ have described their outcomes for FLIP in conjunction with open infrarenal aortic repair in patients with complex aortic dissections. They advocate for the FLIP technique in the setting of significantly narrowed true lumens, suggesting that the chronically thickened dissection septum significantly restricts TEVAR deployment. Other reported techniques for treatment of a significantly narrowed true aortic lumen include septal fenestration^15^ and periscope stenting into the true lumen.^10^ We add another option to the management of complex type B aortic dissection with endovascular bypass of an occluded short-segment true lumen using the FLIP technique.

Limitations of this report include that it is a single case report of a rare clinical circumstance with follow-up imaging through 6 months after the procedure. Despite these limitations, this report describes a technique to address this rare clinical scenario, and the patient was successfully treated without complication, demonstrating initial technical success with evidence of early remodeling of the patient's aneurysmal dissection.

## Conclusions

We present in this report a chronic type B aortic dissection with aneurysmal degeneration that was successfully treated with TEVAR despite a short-segment occluded true lumen that was traversed by aortic septal fenestration and FLIP of the TEVAR devices. More data with long-term follow-up are needed to determine the durability of this type of repair, and selection of patients remains critical. However, this case report suggests that an endovascular approach is possible, safe, and effective for treating these rare lesions.

## References

[1] Hiratzka L.F., Bakris G.L., Beckman J.A., Bersin R.M., Carr V.F., Casey D.E. (2010). 2010 ACCF/AHA/AATS/ACR/ASA/SCA/SCAI/SIR/STS/SVM guidelines for the diagnosis and management of patients with Thoracic Aortic Disease: a report of the American College of Cardiology Foundation/American Heart Association Task Force on Practice Guidelines, American Association for Thoracic Surgery, American College of Radiology, American Stroke Association, Society of Cardiovascular Anesthesiologists, Society for Cardiovascular Angiography and Interventions, Society of Interventional Radiology, Society of Thoracic Surgeons, and Society for Vascular Medicine. Circulation.

[2] Lee W.A., Matsumura J.S., Mitchell R.S., Farber M.A., Greenberg R.K., Azizzadeh A. (2011). Endovascular repair of traumatic thoracic aortic injury: clinical practice guidelines of the Society for Vascular Surgery. J Vasc Surg.

[3] Svensson L.G., Kouchoukos N.T., Miller D.C., Bavaria J.E., Coselli J.S., Curi M.A. (2008). Expert consensus document on the treatment of descending thoracic aortic disease using endovascular stent-grafts. Ann Thorac Surg.

[4] Sachs T., Pomposelli F., Hagberg R., Hamdan A., Wyers M., Giles K. (2010). Open and endovascular repair of type B aortic dissection in the Nationwide Inpatient Sample. J Vasc Surg.

[5] Goodney P.P., Travis L., Lucas F.L., Fillinger M.F., Goodman D.C., Cronenwett J.L. (2011). Survival after open versus endovascular thoracic aortic aneurysm repair in an observational study of the medicare population. Circulation.

[6] Quinones-Baldrich W.J., Saleem T., Oskowitz A. (2018). Infrarenal aortic repair with or without false lumen intentional placement of endografts for hybrid management of complex aortic dissection. J Vasc Surg.

[7] Simring D., Raja J., Morgan-Rowe L., Hague J., Harris P.L., Ivancev K. (2011). Placement of a branched stent graft into the false lumen of a chronic type B aortic dissection. J Vasc Surg.

[8] Kölbel T., Carpenter S.W., Lohrenz C., Tsilimparis N., Larena-Avellaneda A., Debus E.S. (2014). Addressing persistent false lumen flow in chronic aortic dissection: the knickerbocker technique. J Endovasc Ther.

[9] Kölbel T., Lohrenz C., Kieback A., Diener H., Debus E.S., Larena-Avellaneda A. (2013). Distal false lumen occlusion in aortic dissection with a homemade extra-large vascular plug: the candy-plug technique. J Endovasc Ther.

[10] Castro-Ferreira R., Dias P.G., Sampaio S.M., Teixeira J.F., Lachat M. (2018). Simplified hybrid repair with true lumen recycling for retrograde renovisceral perfusion in a complex chronic aortic dissection. J Vasc Surg Cases Innov Tech.

[11] van Bogerijen G.H., Patel H.J., Williams D.M., Yang B., Dasika N.L., Eliason J.L. (2015). Propensity adjusted analysis of open and endovascular thoracic aortic repair for chronic type B dissection: a twenty-year evaluation. Ann Thorac Surg.

[12] Karimi A., Walker K.L., Martin T.D., Hess P.J., Klodell C.T., Feezor R.J. (2012). Midterm cost and effectiveness of thoracic endovascular aortic repair versus open repair. Ann Thorac Surg.

[13] Desai N.D., Burtch K., Moser W., Moeller P., Szeto W.Y., Pochettino A. (2012). Long-term comparison of thoracic endovascular aortic repair (TEVAR) to open surgery for the treatment of thoracic aortic aneurysms. J Thorac Cardiovasc Surg.

[14] Quinones-Baldrich W., Jimenez J.C., DeRubertis B., Moore W.S. (2009). Combined endovascular and surgical approach (CESA) to thoracoabdominal aortic pathology: a 10-year experience. J Vasc Surg.

[15] Barshes N.R., Gravereaux E.C., Semel M., Bolman R.M., Belkin M. (2015). Endovascular longitudinal fenestration and stent graft placement for treatment of aneurysms developing after chronic type B aortic dissection. J Vasc Surg.

